# CSF2 polarized neutrophils and invaded renal cancer cells *in vitro* influence

**DOI:** 10.1515/med-2025-1239

**Published:** 2025-10-28

**Authors:** Yuan Song, Husong Su, Yu Fan, Junfeng Huang, Sheng Xue

**Affiliations:** Department of Urology, The First Affiliated Hospital of Bengbu Medical University, Bengbu, 233004, China; Department of Urology, The First Affiliated Hospital of Bengbu Medical University, 287 Changhuai Road, Bengbu, 233004, China

**Keywords:** renal cancer, neutrophils, polarization, CSF2, PD-L1

## Abstract

**Objective:**

To investigate the role and underlying mechanisms of colony-stimulating factor 2 (CSF2) in the progression of kidney renal clear cell carcinoma (KIRC).

**Methods:**

Transcriptomic and clinical data from The Cancer Genome Atlas were analyzed to assess the correlation between CSF2 expression, clinicopathological features, and patient prognosis. A neutrophil–tumor co-culture system was established to examine the effects of CSF2 on neutrophil polarization, tumor cell proliferation, migration, apoptosis, and autophagy. Protein expression was evaluated by flow cytometry, Western blot, and immunofluorescence. PD-L1 knockout and autophagy inhibitors (3-methyladenine and chloroquine) were used to explore regulatory mechanisms.

**Results:**

CSF2 expression was significantly upregulated in KIRC tissues and was positively associated with advanced tumor stage and poor prognosis. *In vitro*, CSF2 promoted neutrophil polarization toward the tumor-supportive N2 phenotype and enhanced the proliferation and migration of renal cancer cells while inhibiting apoptosis and reactive oxygen species production. Additionally, CSF2 upregulated PD-L1 expression in tumor cells and activated autophagy by increasing LC-3, Beclin1, and ATG7 levels. These effects were reversed by PD-L1 knockout or treatment with the autophagy inhibitor 3-methyladenine.

**Conclusion:**

CSF2 promotes KIRC progression through PD-L1–mediated neutrophil polarization and autophagy activation, representing a potential therapeutic target.

## 1 Introduction

Renal cell carcinoma (RCC) is one of the most prevalent tumors of the urinary system, accounting for approximately 3% of all adult malignancies and over 80% of kidney tumors, with kidney renal clear cell carcinoma (KIRC) being the predominant histological subtype [1]. Granulocyte-macrophage colony-stimulating factor (GM-CSF), also known as CSF2, is a key growth factor in the hematopoietic system that enhances the body’s ability to kill tumor cells by regulating the function of antigen-presenting cells [2]. However, the expression levels and prognostic significance of CSF2 vary across solid malignancies and remain controversial [3,4]. Recent studies have identified CSF2 as a key gene associated with prognosis and immune regulation in KIRC. It was included in a nine-gene apoptosis-related prognostic signature with strong predictive power for overall survival [5]. Moreover, CSF2 was also part of an immune-related gene signature that predicted immunotherapeutic efficacy and reflected the tumor immune microenvironment, underscoring its value in personalized therapy [6]. Functional analyses further suggest that CSF2 inhibits tumor cell proliferation, infiltration, and migration, indicating its potential as a therapeutic target [7]. Studies have shown that elevated CSF2 levels in several malignancies have been associated with poor prognosis [8,9]. Chronic inflammation is a well-established driver of tumorigenesis and progression, influencing tumor development, invasion, metastasis, and therapy response [10,11]. Moreover, tumor-infiltrating neutrophils play a key role in renal cancer progression, lung metastasis, and angiogenesis [12]. Recent large-scale informatics studies have highlighted a global shift in oncological research focus toward immunotherapy, particularly neoadjuvant strategies, revealing emerging interest in biomarkers, tumor microenvironment, and immune checkpoint regulation as pivotal determinants of therapeutic outcomes [13].

The tumor microenvironment (TME) of RCC involves complex immune cell infiltration, including T cells, macrophages, and neutrophils, which together shape tumor progression and immune response. T cells exhibit stage-specific infiltration patterns, with CD4^+^ peaking in Stage 3 and CD8^+^ increasing through Stage 4, reflecting their dual role in surveillance and progression [14]. Tumor-associated macrophages, particularly M2-polarized subtypes, promote immunosuppression, angiogenesis, and invasion [15]. Neutrophils are increasingly recognized for their pro-tumor roles, with high neutrophil-to-lymphocyte ratios linked to poor prognosis and increased infiltration observed in tumors post-immunotherapy, suggesting a role in resistance [16,17]. Neutrophils and macrophages (particularly the M2 subtype) work synergistically in this environment, promoting an immunosuppressive milieu that aids tumor escape from immune surveillance [18]. Therefore, the TME in RCC is complex, involving various immune cells that both support tumor progression and pose challenges for immunotherapy. Neutrophils play a significant role in the TME of RCC, where their polarization into pro-tumor N2 phenotypes promotes tumor progression and immune suppression. In KIRC, neutrophils are recruited by chemokines such as CXCL2, which not only mediate their infiltration but also drive their polarization toward an immunosuppressive N2 phenotype, promoting angiogenesis, immune evasion, and tumor progression [19]. Moreover, interactions between neutrophils and M2 macrophages further amplify immunosuppression and support tumor growth [20]. These findings suggest that targeting neutrophil polarization could be a potential strategy to improve RCC treatment outcomes.

Studies suggest that programmed cell death ligand 1 (PD-L1), a key immune checkpoint protein, is involved in this process by regulating neutrophil polarization. For instance, neutrophil extracellular traps ejected by activated neutrophils exert immunosuppressive functions via programmed death 1 (PD-1)/PD-L1 [21]. Autophagy, a cellular process responsible for degrading and recycling cellular components, is intricately linked to neutrophil function and polarization. Autophagy in neutrophils regulates their survival, activation, and polarization. It plays a pivotal role in the N2 polarization of neutrophils by modulating inflammatory responses and metabolic pathways. Studies have shown that autophagy in neutrophils can enhance their survival and pro-tumor functions by facilitating the production of reactive oxygen species (ROS), which are essential for their antimicrobial activity and polarization [22]. Additionally, autophagy-induced neutrophils contribute to cancer progression by increasing the secretion of inflammatory factors and promoting tumor cell migration [23]. Despite these findings, the specific role of CSF2 in modulating neutrophil polarization within the renal cancer microenvironment remains unclear. Building on these insights, we hypothesize that CSF2 may induce N2 polarization of neutrophils through the PD-L1 pathway, thereby driving tumor progression in RCC. By enhancing neutrophil polarization and promoting an immunosuppressive environment, CSF2 could potentially contribute to immune evasion and cancer progression. This study aims to investigate the role of CSF2 in neutrophil polarization in RCC, with the potential to uncover novel therapeutic targets. The significance of this research lies in its potential to elucidate key mechanisms of tumor progression and metastasis and to support the development of innovative immunotherapeutic strategies.

## 2 Materials and methods

### 2.1 Bioinformatic analysis

Transcriptomic data of KIRC were obtained from The Cancer Genome Atlas (TCGA, https://portal.gdc.cancer.gov; 202208). RNA-sequencing (RNA-seq) data processed using the STAR pipeline were downloaded in fragments per kilobase of transcript per million mapped reads (FPKM) format. FPKM values were converted to transcripts per million (TPM) for cross-sample normalization. The dataset comprised 613 tumors and 72 adjacent normal tissue samples. Clinical data were available for 537 patients, and clinical annotations were linked to all 613 RNA-seq samples. To ensure sample independence, nine duplicate RNA-seq samples from the same patients were excluded. Samples lacking clinical data or classified as technical duplicates were also removed to enhance the reliability of statistical inference. Gene expression data were log_2_-transformed [log_2_(TPM + 1)] to normalize distributions and reduce heteroscedasticity. CSF2 expression (ENSG00000164400.6) was extracted for downstream analysis. Comparisons between tumor and normal tissues were conducted using the Wilcoxon rank-sum test, implemented with the stats (v4.2.1) and car (v3.1-0) packages in R (v4.2.1). Data visualization was performed using ggplot2 (v3.4.4). Potential confounders, including sample duplication and missing clinical annotations, were rigorously addressed during data preprocessing.

### 2.2 Cell culture

Human renal cancer cell line 786-O (CL-0010) was procured from Wuhan Pricella Biotechnology Co., Ltd. 786-O renal cancer cell line is known for its resistance to chemotherapy and immunotherapy, making it suitable for investigating potential therapeutic strategies and understanding tumor progression and metastasis mechanisms [24]. Cells were cultured in high-glucose Dulbecco’s Modified Eagle Medium (Cat. No.: D5796, Sigma-Aldrich), supplemented with 10% premium fetal bovine serum (FBS; Cat. No.: 16000044, Thermo Fisher Scientific) and 1% penicillin-streptomycin (Cat. No.: 15140122, Thermo Fisher Scientific). Cultures were maintained in a humidified incubator at 37°C with 5% CO_2_. Upon reaching 80% confluency, cells were harvested using 0.25% trypsin-ethylenediaminetetraacetic acid (Cat. No.: 25200056, Thermo Fisher Scientific) for subsequent experiments.

HL-60 cells (Cat. No.: CL-0110) were purchased from Wuhan Pricella Biotechnology Co., Ltd., and can be induced to differentiate into neutrophil-like cells. HL-60 cells are widely used as a model for neutrophil differentiation due to their ability to mimic neutrophil-like behavior upon differentiation, providing an efficient and reproducible system to study neutrophil functions [25,26]. HL-60 cells were cultured in Iscove's modified Dulbecco's medium (Procell, Wuhan, China, PM150510C) supplemented with 20% FBS and 1% penicillin-streptomycin. Differentiation of HL-60 cells into neutrophil-like cells was achieved using a standard protocol involving 1.25% dimethyl sulfoxide (Sigma-Aldrich) for 4 days, as previously described [27]. Differentiation efficacy was assessed by flow cytometry using cluster of differentiation 11b (CD11b) as a neutrophil marker, achieving an 80% CD11b^+^ population [28]. After differentiation, neutrophil-like cells were co-cultured with 786-O cells at a 1:1 ratio in medium containing 20 ng/mL CSF2 (Peprotech, Cat No.: 300-03) for 5 days at 37°C and 5% CO_2_.

### 2.3 PD-L1 knockout

To investigate the role of PD-L1 in CSF2-mediated effects, PD-L1 knockout 786-O cells were generated using CRISPR/Cas9 gene-editing technology. Cells were transfected with a PD-L1-targeting CRISPR/Cas9 plasmid (Santa Cruz Biotechnology, sc-401626, sgRNA sequence: GCGAGCTAGCGAGATACT) using Lipofectamine 3000 (Thermo Fisher Scientific, Cat No.: L3000008) following the manufacturer’s protocol. After 48 h, cells were selected with puromycin (2 μg/mL) for 7 days. Knockout efficiency was confirmed by Western blot using an anti-PD-L1 antibody (Abcam, Cat No.: ab213524). Knockout clones were expanded and used in subsequent co-culture and functional assays under the same conditions as the wild-type cells. To assess autophagy, co-culture cells were treated with 3-methyladenine (3-MA, 5 mM, Selleck, Cat No.: S2767) to inhibit early-stage autophagy [29] and chloroquine (CQ, 10 μM, Yeasen, Cat No.: 4608) for 24 h to inhibit late-stage autophagy [30]. Untreated cells served as the negative control group (Control).

### 2.4 Flow cytometry analysis

Flow cytometry analysis of CD11b, PD-L1, CD54, CD86, CD163, and CD206 were assessed on neutrophil-like HL-60 cells. Cells were collected from the culture medium and washed twice with PBS to remove residual medium. Subsequently, the cells were resuspended in PBS containing 0.5% bovine serum albumin (BSA, Sigma-Aldrich, Cat No.: A9418) and adjusted to a concentration of 1 × 10^6^ cells/mL. Cells were incubated separately with fluorescein isothiocyanate (FITC)-conjugated CD11b antibody (BioLegend, Cat No.: 101206), phycoerythrin (PE)-conjugated PD-L1 antibody (BD Biosciences, Cat No.: 558065), APC-conjugated CD54 (BioLegend, Cat No.: 353108), FITC-conjugated CD86 (BioLegend, Cat No.: 305404), PE-conjugated CD163 (BioLegend, Cat No.: 333606), APC-conjugated CD206 (BioLegend, Cat No.: 321110) at 4°C in the dark for 30 min. For each staining condition, isotype control antibodies (FITC- or PE-conjugated, matched to the host species and subclass) were included to determine non-specific binding. Isotype-matched controls were included for each fluorochrome. For ROS detection, cells were incubated with 2′,7′-dichlorofluorescein diacetate (DCFH-DA, Beyotime, Cat No.: S0033S) at a final concentration of 10 μM in serum-free medium. The cells were maintained at 37°C for 20 min in the dark with gentle agitation every 5 min. After incubation, cells were washed three times with serum-free medium to remove excess probe. The 2′,7′-dichlorofluorescein fluorescence intensity was measured, reflecting intracellular ROS levels. Stained cells were analyzed using a BD FACSCalibur flow cytometer, acquiring at least 10,000 events per sample. Data analysis was performed using FlowJo software (v10, BD Biosciences).

### 2.5 Apoptosis detection by flow cytometry

Cells were harvested and centrifuged at 300 × *g* for 5 min at 4°C. For apoptosis detection, the cells were resuspended in 1× binding buffer (Cat No.: 556454, BD Biosciences) and stained with Annexin V-APC (Cat No.: 550474, BD Biosciences) according to the manufacturer’s instructions. Following incubation, the cells underwent a second centrifugation at 300 × *g* for 5 min at 4°C. Subsequently, the supernatant was discarded, and the cells were resuspended in binding buffer with the addition of propidium iodide (PI) solution (Cat No.: P4864, Sigma-Aldrich) to a final concentration of 5 µg/mL. The stained cells were then transferred to flow cytometry tubes and analyzed using a BD FACSCanto II flow cytometer (BD Biosciences). Data acquisition and analysis were conducted using FlowJo software, allowing the quantification of apoptotic cells by distinguishing Annexin V-positive/PI-negative (early apoptosis) from Annexin V-positive/PI-positive (late apoptosis) populations.

### 2.6 CCK-8 assay

During the co-culture period, cell proliferation was assessed daily using the Cell Counting Kit-8 (CCK-8, Dojindo, Cat No.: CK04). On days 1, 2, 3, 4, and 5 of culture, 10 µL of CCK-8 reagent was added to each well, followed by incubation at 37°C and 5% CO_2_ for 2 h. Absorbance was measured at 450 nm using a microplate reader (Molecular Devices, SpectraMax M5) to quantify cell proliferation.

### 2.7 Transwell assay

Cell migration was assessed using Transwell chambers (Corning, Cat No.: 3422). Serum-free medium (100 µL) was added to the upper chamber, and 150,000 cells in 100 µL were seeded. The lower chamber contained 600 µL of medium with 25% FBS as a chemoattractant. Subsequently, 100 µL of the cell suspension was added to each upper chamber. The plates were then incubated in a humidified atmosphere with 5% CO_2_ at 37°C for 24 h. Following incubation, the non-migrated cells were removed from the upper surface of the membrane using a cotton swab. The migrated cells on the lower surface were fixed with 4% paraformaldehyde (Cat No.: 158127, Sigma-Aldrich) for 15 min, stained with 0.1% crystal violet (Cat No.: C0775, Sigma-Aldrich) for 10 min, and washed three times with distilled water. The membranes were then carefully removed, mounted on slides, and imaged under a microscope (Nikon Eclipse Ti, Nikon Instruments Inc.) at 200× magnification to capture representative fields for analysis. The images were retained for quantification of cell migration.

### 2.8 Western blot

The protein concentrations were determined using the Bicinchoninic Acid (BCA) Protein Assay Kit (AP12L025, Shanghai Life-iLab Biotech Co., Ltd). Diluted protein standard solutions and BCA working solution were added to a 96-well microplate according to the manufacturer’s instructions. Absorbance values were measured at 562 nm using a microplate reader (Cat No.: 168-1000, Bio-Rad). A standard curve was constructed to calculate the protein concentrations of the samples. For Western blot analysis, equal amounts of protein (20 µg per lane) were separated by SDS-PAGE on a 10% polyacrylamide gel and transferred to polyvinylidene difluoride membranes (Cat No.: IPVH00010, Millipore). The membranes were blocked with 5% skim milk powder in Tris-buffered saline (TBS; 10 mM Tris, 150 mM NaCl, pH 7.4) at room temperature for 1 h with gentle shaking. After blocking, the membranes were washed three times with TBS containing 0.1% Tween-20 (TBST). The membranes were then incubated with PD-L1 (Abcam, ab213524, 1:1,000), TNF-related apoptosis-inducing ligand (TRAIL) (Abcam, ab42121, 1:10,000), Prokineticin 2 (PK2, also known as Bv8) (Abcam, ab87360, 1:5,000), microtubule-associated protein 1A/1B-light chain 3 (LC-3) (Abcam, ab192890, 1:2,000), Autophagy-related 7 (ATG7) (Abcam, ab52472, 1:100,000), Beclin 1 (Abcam, ab207612, 1:2,000) and GAPDH (Abcam, ab8245, 1:5,000) primary antibodies in TBST at 4°C overnight with gentle shaking. GAPDH was used as the internal loading control. The next day, the membranes were washed three times with TBST and incubated with HRP-conjugated secondary antibodies (Abcam, ab6721, 1:10,000) at room temperature for 30 min with gentle shaking. Following three additional washes with TBST, the protein bands were detected using an enhanced chemiluminescence kit (Cat No.: RPN2232, GE Healthcare) and imaged using a ChemiDoc MP Imaging System (Bio-Rad). Relative band intensities were normalized to GAPDH and quantified using ImageJ software (NIH, USA).

### 2.9 Immunofluorescence staining

Cells were fixed with 4% paraformaldehyde solution for 20 min, followed by permeabilization with 0.1% Triton X-100 (Sigma-Aldrich, Cat No.: T8787) for 10 min. The cells were then blocked with 1% BSA (Sigma-Aldrich, Cat No.: A2153) in PBS for 30 min to prevent nonspecific binding. Overnight incubation at 4°C was performed using a primary antibody against LC-3 (Abcam, Cat No.: ab192890) diluted at 1:200. The next day, cells were incubated with an Alexa Fluor 488-conjugated secondary antibody (Thermo Fisher, Cat No.: A11029) at a dilution of 1:500 for 1 h in the dark. Nuclei were stained for 15 min using 4′,6-diamidino-2-phenylindole (Thermo Fisher, Cat No.: D1306). Finally, images were captured using a fluorescence microscope (Nikon, Eclipse Ti2) to analyze the formation of LC-3 puncta.

### 2.10 Statistical analysis

All experiments were independently conducted in triplicate. Data are presented as means ± standard deviation (SD). Statistical significance was determined using an unpaired two-tailed Student’s *t*-test for comparisons between two groups, and one-way ANOVA for comparisons among three or more groups, with a significance threshold set at *P* < 0.05. Data processing and statistical analyses were performed using GraphPad Prism software (v 9.0, GraphPad Software, San Diego, CA) and R software (v4.2.1). For the analysis of TCGA-KIRC RNA-seq and clinical data, gene expression values were transformed as log_2_(TPM + 1). Kruskal–Wallis tests were used to compare CSF2 expression across multiple pathological stages and T stages, while Wilcoxon rank-sum tests were applied for binary comparisons of N and M stages. Kaplan–Meier survival curves were constructed for overall survival analysis and compared using the log-rank test. R packages including ggplot2 (v3.4.4), stats (v4.2.1), and car (v3.1-0) were used for statistical testing and visualization.


**Ethics approval:** This study did not require ethical board approval because it did not perform human or animal trials.
**Consent to participate:** Not applicable.
**Patient consent for publication:** Not applicable for no human trials.

## 3 Results

### 3.1 High CSF2 expression correlates with disease progression and poor prognosis in KIRC

**Figure 1:**
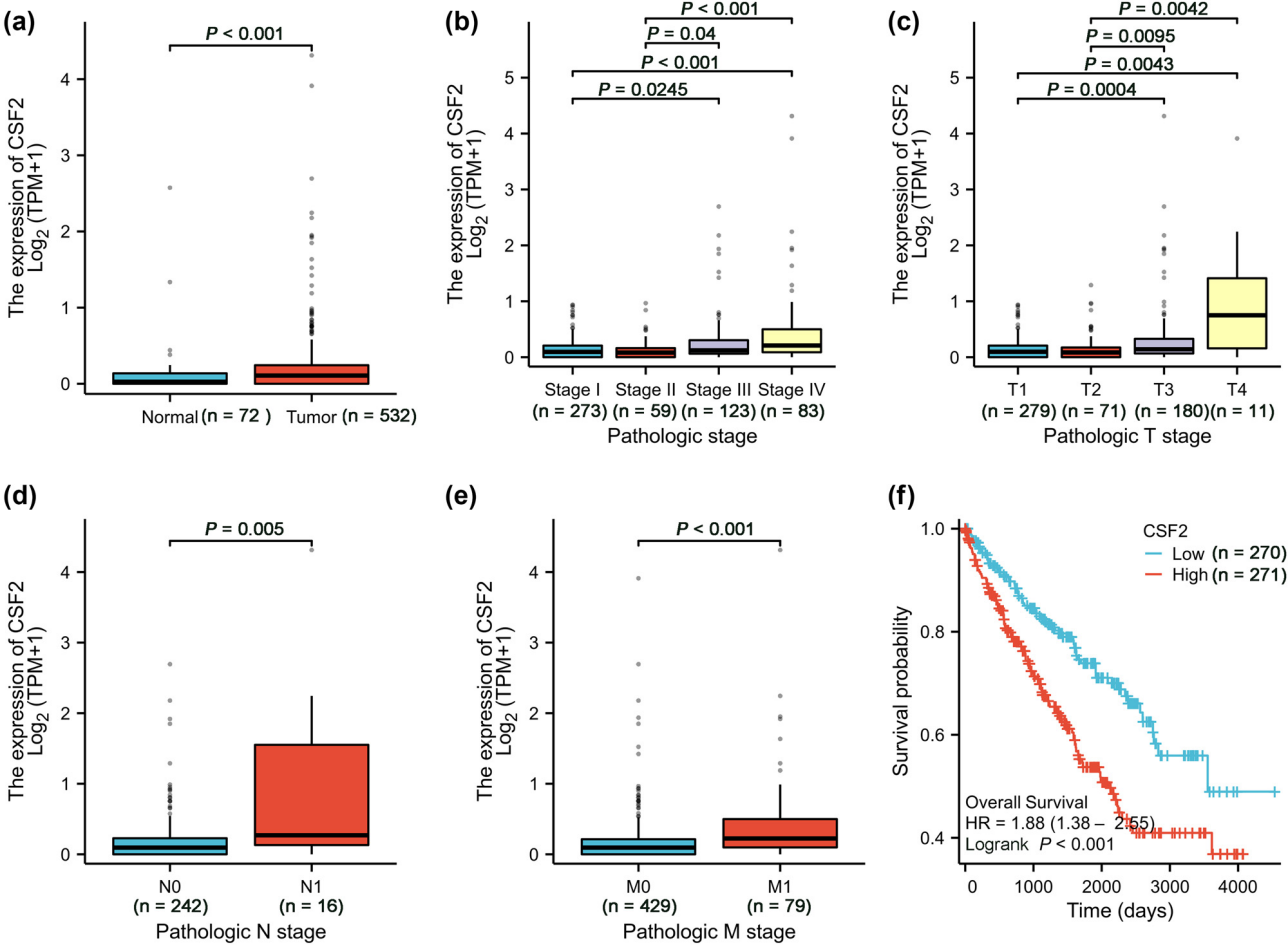
High CSF2 expression correlates with disease progression and poor prognosis in KIRC. (a) Comparison of CSF2 expression between normal and tumor tissues (Wilcoxon rank sum test). (b) CSF2 expression in different pathological stages (Stage I to Stage IV) (Kruskal–Wallis test). (c) CSF2 expression in different pathological T stages (T1 to T4) (Kruskal–Wallis test). (d) CSF2 expression in different pathological N stages (N0 and N1) (Wilcoxon rank sum test). (e) CSF2 expression in different pathological M stages (M0 and M1) (Wilcoxon rank sum test). (f) Overall survival comparison between high and low CSF2 expression groups (Log-rank test). KIRC: Kidney renal clear cell carcinoma.

Analysis of KIRC data from TCGA revealed that CSF2 expression is significantly higher in KIRC tissues compared to adjacent normal tissues (Figure 1a). This differential expression suggests that CSF2 may play an active role in tumorigenesis or reflect alterations in the tumor microenvironment. Furthermore, CSF2 expression increased progressively across pathological stages, with higher expression levels observed in advanced stages (Stage IV) compared to early stages (Stage I) (Figure 1b). This pattern implicates CSF2 in tumor progression, potentially by fostering inflammation or modulating immune responses that favor tumor growth and metastasis. Similarly, CSF2 expression increases with higher pathological T stages (T1–T4), with higher T stages showing significantly higher expression than lower T stages (Figure 1c). Since higher T stage reflects greater tumor size and invasion, this trend supports the idea that CSF2 may contribute to tumor aggressiveness, perhaps by influencing cell proliferation or extracellular matrix remodeling. Patients with lymph node metastasis (N1 stage) exhibited significantly higher CSF2 expression than those without nodal involvement (N0 stage) (Figure 1d), indicating a potential role for CSF2 in facilitating lymphatic dissemination. Additionally, CSF2 expression was significantly higher in patients with distant metastases (M1 stage) than in those without (M0 stage) (Figure 1e). Kaplan–Meier survival analysis further demonstrated that patients with high CSF2 expression had significantly shorter overall survival than those with low expression, supporting its prognostic relevance in KIRC (Figure 1f).

### 3.2 CSF2 promotes N2 polarization of neutrophils and enhances proliferation and migration of co-cultured renal cancer cells

**Figure 2:**
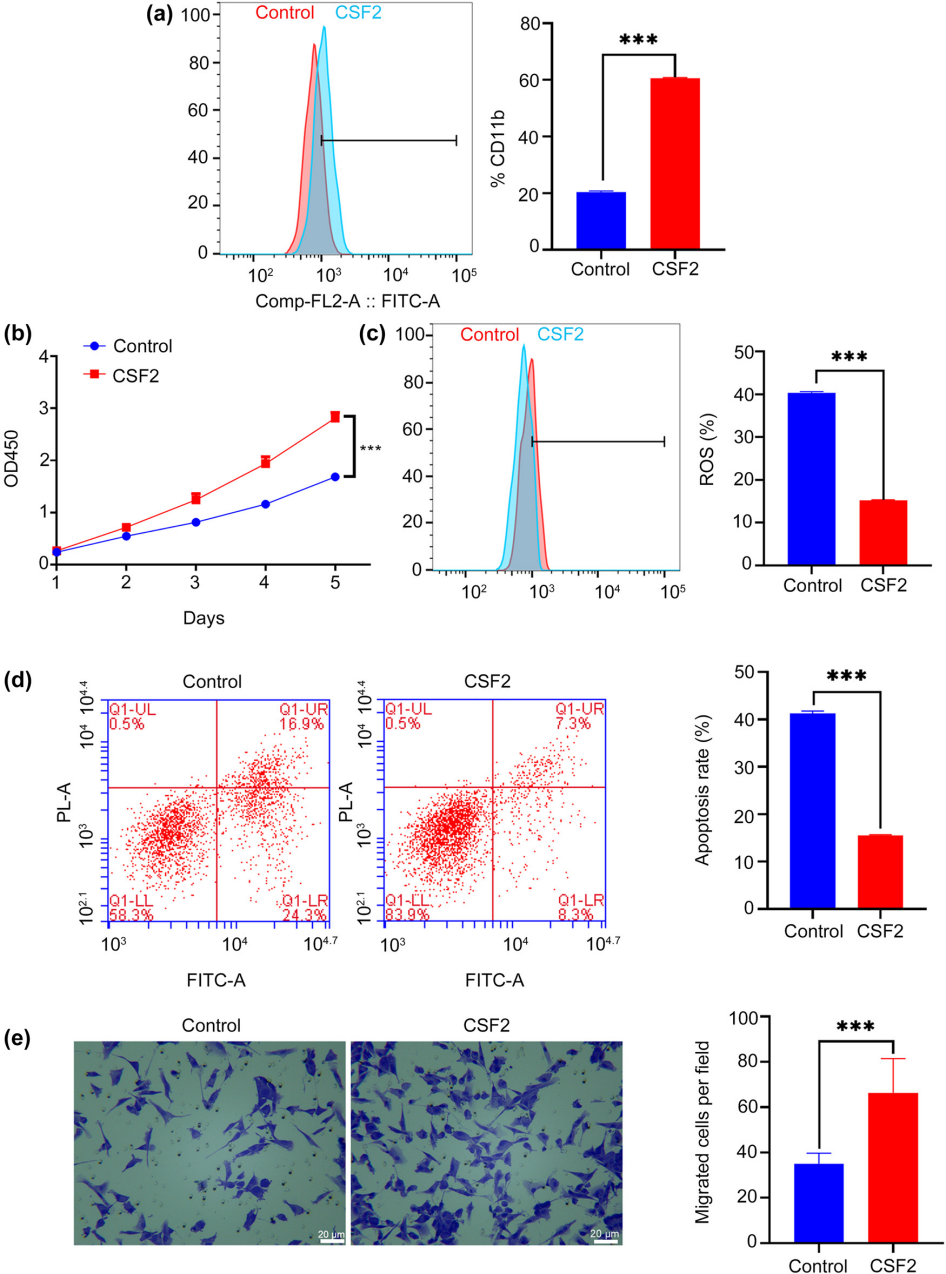
CSF2 promotes N2 polarization of neutrophils and enhances the proliferation and migration of co-cultured renal cancer cells. (a) Measurement of CD11b expression on neutrophils by flow cytometry. (b) Proliferation assay of renal cancer cells in co-culture with neutrophils. (c) Measurement of reactive oxygen species (ROS) levels in renal cancer cells by flow cytometry. (d) Apoptosis assay of renal cancer cells in co-culture with neutrophils using flow cytometry. (e) Migration assay showing the number of migrated renal cancer cells in co-culture with neutrophils (200×); Data are presented as mean ± SD (*n* = 3). Statistical significance was assessed using Student’s *t*-test. Compared with control group, ^***^ *P* < 0.001.

CSF2 treatment significantly increases CD11b expression in neutrophils compared to the control group (*P* < 0.001, Figure 2a). The proliferation rate of renal cancer cells co-cultured with CSF2-treated neutrophils is significantly higher over 5 days compared to the control group (*P* < 0.001, Figure 2b). The level of ROS in renal cancer cells co-cultured with CSF2-treated neutrophils is significantly lower than in the control group (*P* < 0.001, Figure 2c). Given that excessive ROS can induce tumor cell apoptosis, this reduction may reflect a protective effect provided by N2-polarized neutrophils, helping cancer cells evade oxidative stress [31]. The apoptosis rate of renal cancer cells co-cultured with CSF2-treated neutrophils is significantly lower than in the control group (*P* < 0.001, Figure 2d). This further supports the role of CSF2-induced neutrophils in creating a pro-survival microenvironment for renal cancer cells, possibly by secreting anti-apoptotic cytokines or modulating cell signaling pathways. The number of migrated renal cancer cells in co-culture with CSF2-treated neutrophils is significantly higher than in the control group (*P* < 0.001, Figure 2e). This implies that CSF2-activated neutrophils enhance tumor cell motility, potentially through remodeling of the extracellular matrix or secretion of chemotactic factors that facilitate metastasis.

### 3.3 CSF2-induced N2 polarization is partially mediated by tumor-derived PD-L1

**Figure 3:**
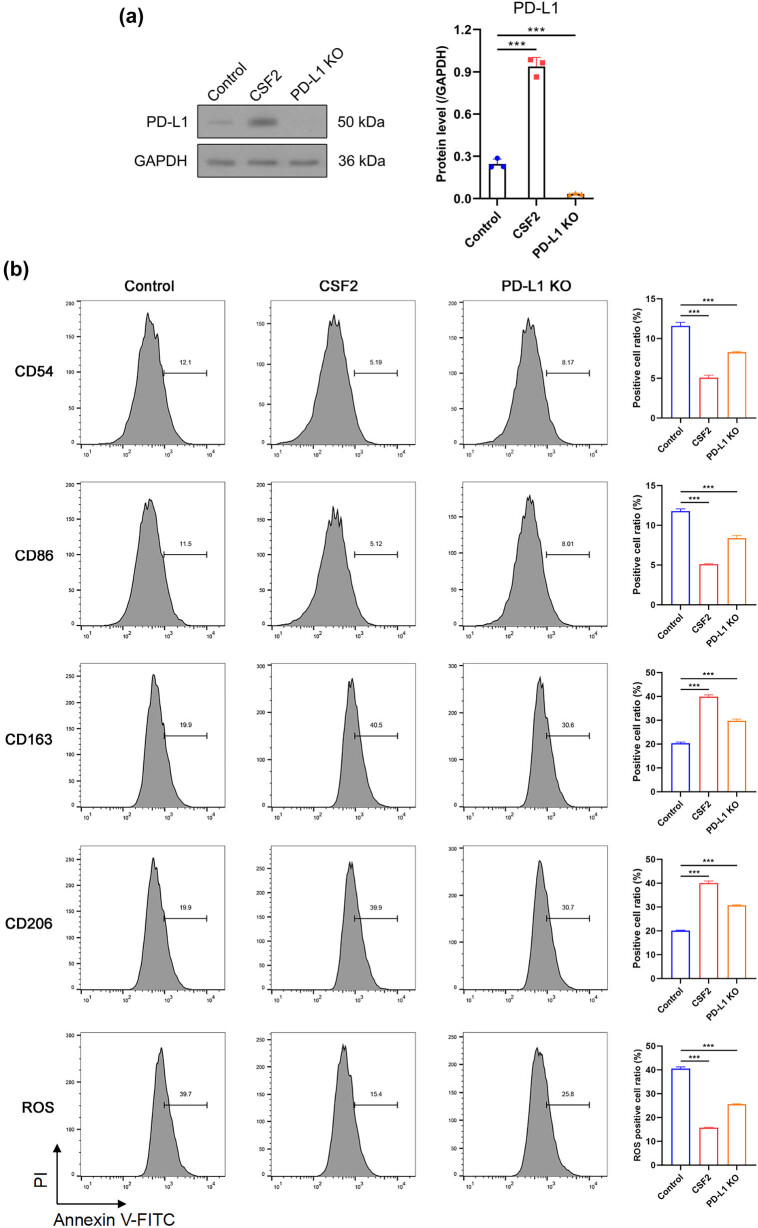
CSF2-induced N2 polarization is partially mediated by tumor-derived PD-L1. (a) Western blot analysis of PD-L1 protein expression in renal cancer cells with or without CSF2 stimulation and PD-L1 knockout. (b) Flow cytometry analysis of neutrophil polarization markers (CD163, CD206, CD54, CD86) and intracellular ROS levels under CSF2 treatment and co-culture with PD-L1 knockout or control tumor cells. Data are presented as mean ± SD (*n* = 3). Statistical significance was assessed using one-way ANOVA. Compared with control group, ^***^ *P* < 0.001.

To confirm the efficiency of PD-L1 knockout, Western blot analysis demonstrated that PD-L1 protein levels were almost undetectable in the PD-L1 knockout cells (Figure 3a). Flow cytometry revealed that CSF2 treatment increased N2 markers (CD163, CD206) and decreased N1 markers (CD54, CD86) in neutrophils (*P* < 0.001). The CD54 (high) neutrophils are a kind of reverse-transmigrated neutrophils characterized by proinflammatory phenotype [32] and CD163 and CD206 as markers of the immunosuppressive N2 phenotype [33]. when neutrophils were co-cultured with PD-L1 knockout renal cancer cells, the CSF2-induced shift toward the N2 phenotype was partially attenuated, with decreased CD163/CD206 expression and a partial recovery of CD54/CD86 levels compared to co-culture with wild-type tumor cells. In parallel, CSF2-treated neutrophils significantly reduced intracellular ROS levels in co-cultured renal cancer cells, indicating a shift toward a less oxidative, pro-survival microenvironment. Importantly, when co-cultured with PD-L1 knockout renal cancer cells, both N2 marker induction and ROS suppression were partially reversed, suggesting that tumor-derived PD-L1 is required for the full execution of the CSF2-induced immunosuppressive program in neutrophils (Figure 3b). These findings suggest a CSF2–PD-L1 axis that modulates neutrophil reprogramming and oxidative stress responses in favor of tumor survival.

### 3.4 CSF2-treated neutrophils affect the PD-L1 pathway proteins in co-cultured renal cancer cells

**Figure 4:**
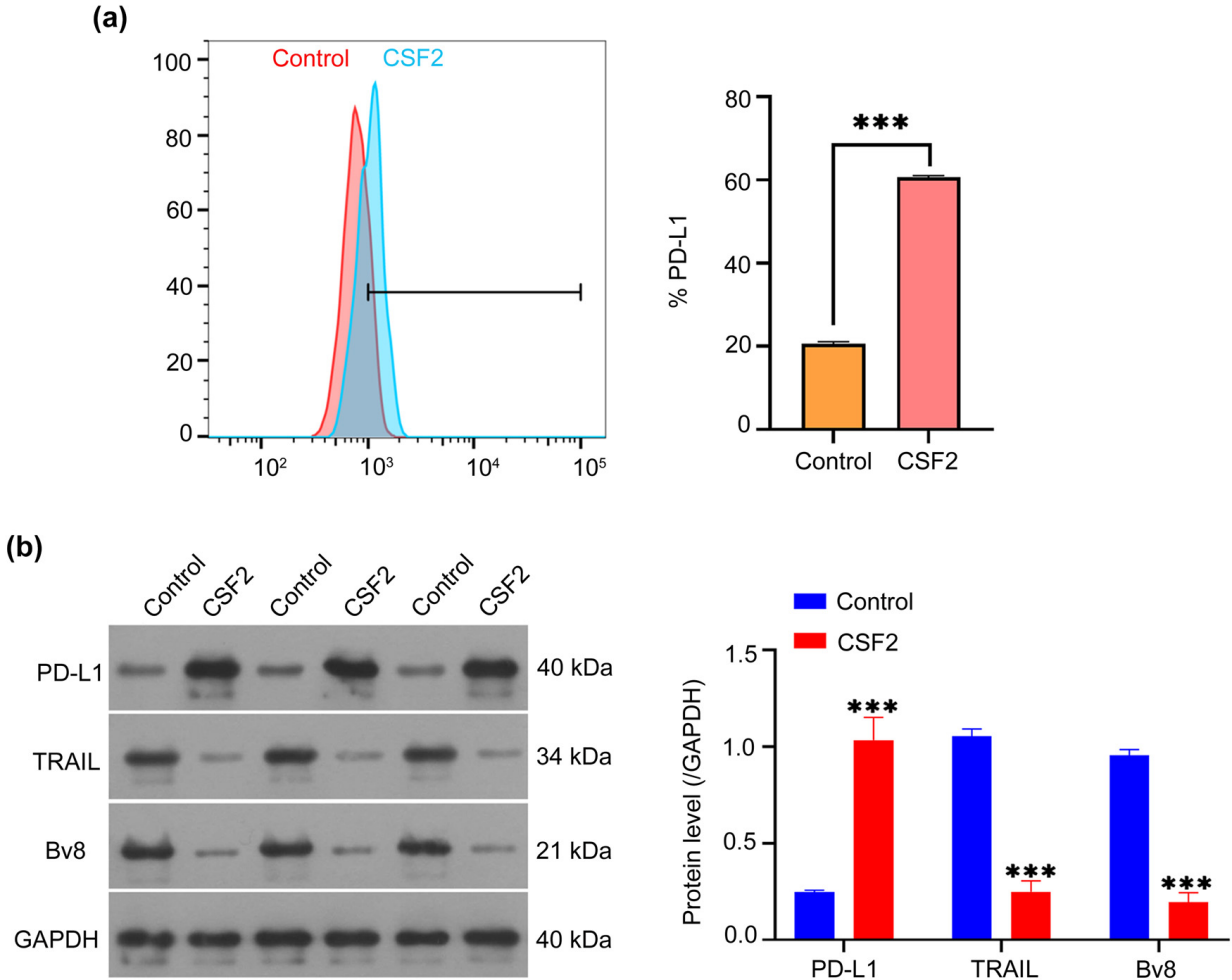
CSF2-treated neutrophils affect the PD-L1 pathway proteins in co-cultured renal cancer cells. (a) Measurement of PD-L1 expression on co-cultured renal cancer cells by flow cytometry. Data are presented as mean ± SD (*n* = 3). Statistical significance was assessed using Student’s *t*-test. Compared with the control group, ^***^ *P* < 0.001. (b) Western blot analysis of PD-L1, TRAIL, and Bv8 protein levels in co-cultured renal cancer cells. Data are presented as mean ± SD (*n* = 3). Statistical significance was assessed using one-way ANOVA. Compared with the control group, ^***^ *P* < 0.001. PD-L1: Programmed death-ligand 1; TRAIL: TNF-related apoptosis-inducing ligand; Bv8: Prokineticin 2.

Flow cytometry and Western blot analyses revealed that CSF2-treated neutrophils upregulated PD-L1 expression and downregulated TRAIL and Bv8 in co-cultured renal cancer cells (*P* < 0.001, Figure 4a and b). The protein levels of PD-L1 are significantly increased, while the levels of TRAIL and Bv8 are significantly decreased in renal cancer cells co-cultured with CSF2-treated neutrophils compared to the control group (*P* < 0.001, Figure 4b). This suggests that CSF2-primed neutrophils facilitate immune evasion by inducing PD-L1, while suppressing apoptotic and pro-angiogenic signaling pathways.

### 3.5 Autophagy activation by CSF2-treated neutrophils depends on PD-L1

**Figure 5:**
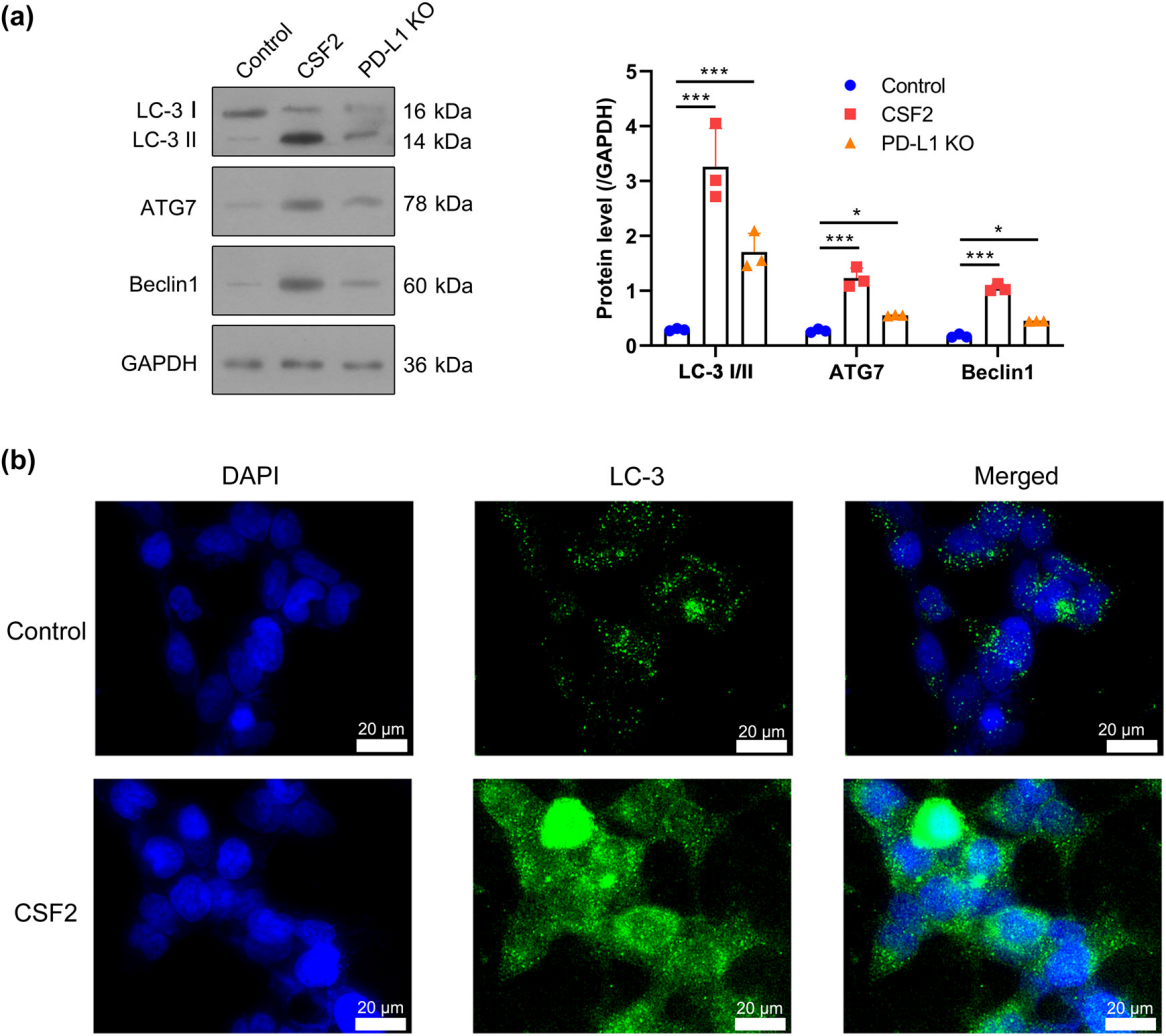
Autophagy activation by CSF2-treated neutrophils depends on PD-L1. (a) Western blot analysis of LC-3, ATG7, and Beclin1 protein levels in co-cultured renal cancer cells. Data are presented as mean ± SD (*n* = 3). Statistical significance was assessed using one-way ANOVA. Compared with the control group, ^***^ *P* < 0.001. (b) Immunofluorescence staining of LC-3 in renal cancer cells with DAPI as a nuclear counterstain. LC-3: Microtubule-associated protein 1A/1B-light chain 3; ATG7: Autophagy-related 7; DAPI: 4′,6-diamidino-2-phenylindole.

Western blot analysis demonstrated that CSF2 treatment increased autophagy-related proteins LC-3 II/I, ATG7, and Beclin1 in co-cultured renal cancer cells (*P* < 0.001). PD-L1 knockout reversed autophagy induced by CSF2 treatment, and the expression of related proteins LC-3 II/I (*P* < 0.001), ATG7, and Beclin1 was significantly decreased (*P* < 0.05), suggesting that PD-L1 is involved in CSF2-mediated autophagy regulation (Figure 5a). Immunofluorescence confirmed increased LC-3 puncta formation, indicating enhanced autophagy (Figure 5b). These results suggest that PD-L1 mediates CSF2-driven autophagy, contributing to tumor cell survival and chemoresistance.

### 3.6 CSF2 promotes N2 polarization of neutrophils, and the migration-promoting effect on co-cultured renal cell carcinoma is reversed by 3-MA

**Figure 6:**
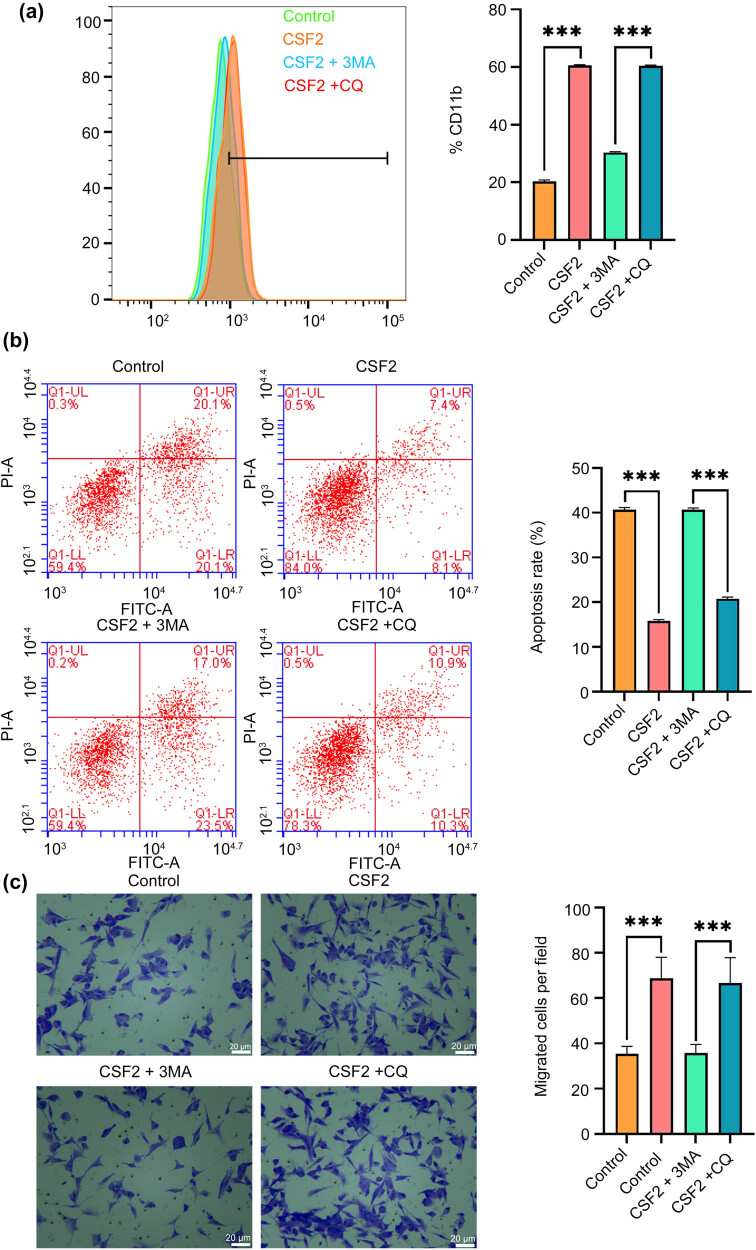
CSF2 promotes N2 polarization of neutrophils, and the migration-promoting effect on renal cell carcinoma is reversed by 3-MA. (a) Measurement of CD11b expression on neutrophils by flow cytometry. (b) Apoptosis assay of renal cancer cells in co-culture with neutrophils using flow cytometry. (c) Migration assay showing the number of migrated renal cancer cells in co-culture with neutrophils (200×). Data are presented as mean ± SD (*n* = 3). Statistical significance was assessed using Student’s *t*-test. Compared with the control group, ^***^ *P* < 0.001. Cluster of differentiation 11b; 3-MA: 3-Methyladenine; CQ: Chloroquine.

CSF2 treatment significantly increases CD11b expression in neutrophils compared to the control group, and this effect is significantly reversed by 3-MA but not by CQ (*P* < 0.001, Figure 6a). The apoptosis rate of renal cancer cells co-cultured with CSF2-treated neutrophils is significantly lower than in the control group, but this effect is partially reversed by 3-MA (*P* < 0.001, Figure 6b). The number of migrated renal cancer cells in co-culture with CSF2-treated neutrophils is significantly higher than in the control group, and this migration-promoting effect is significantly reversed by 3-MA but not by CQ (*P* < 0.001, Figure 6c). These results indicate that CSF2-induced N2 polarization of neutrophils drives tumor progression through autophagy-dependent suppression of apoptosis and enhancement of migration, highlighting the critical role of autophagy in shaping an immunosuppressive niche for renal cancer metastasis. CSF2-treated neutrophils upregulate PD-L1 and suppress apoptosis-angiogenesis signaling in renal cancer cells.

## 4 Discussion

Renal cell carcinoma, a common malignant tumor of the urinary system, is difficult to detect early and is often treated surgically, but recurrence is unfortunately common [34]. Early-stage differentiation between benign and malignant renal masses is also challenging [35]. Once the disease progresses to advanced stages, systemic therapy becomes the mainstay of treatment. Nevertheless, existing immune and targeted therapies have not yielded optimal clinical outcomes, with drug resistance representing a major obstacle [36]. Although recent advancements involving probiotics, HIF-2α inhibitors, and stereotactic radiotherapy have shown promise in prolonging survival in advanced cases, the identification of novel therapeutic targets remains an urgent need [37]. Previous studies have shown that CSF2 can affect the progression of malignancy [3,38]. Initially discovered in 1977, CSF2 was identified for its role in stimulating colony formation in granulocytes and macrophages [39]. Since then, the immune function of CSF2 has been extensively studied, and its role in anti-tumor immunity is well-established in research and clinical applications [40]. However, its specific influence on the tumor microenvironment and the mechanisms through which it may promote tumor progression remain insufficiently elucidated. Building upon its reported pro-tumorigenic functions in gastric and colorectal cancers [41], this study selected CSF2 as a focal gene for investigation.

Recent studies have underscored the clinical relevance of CSF2 in KIRC, showing that its elevated expression is consistently associated with poor prognosis and an immunosuppressive tumor microenvironment. For instance, CSF2 was incorporated into a natural killer cell-related prognostic signature that stratifies KIRC patients by risk and predicts differential sensitivity to immune checkpoint inhibitors and chemotherapeutic agents, suggesting its potential utility in guiding personalized therapy [42]. Similarly, a lymphangiogenesis-related gene model including CSF2 was found to correlate with T-cell exhaustion and immunosuppressive cell infiltration, highlighting its role in shaping the immune microenvironment and informing immunotherapeutic response [43]. This study revealed that CSF2 expression was significantly higher in cancer tissues than in adjacent normal tissues and was positively correlated with Stage, Pathologic_T, Pathologic_N, and Pathologic_M, as well as being associated with poorer prognosis. This result is also in line with the findings of other scholars in breast cancer, head and neck cancer, bladder cancer, and other tumors [44]. These findings suggest that targeting CSF2 or its downstream pathways may enhance anti-tumor immunity and improve clinical outcomes in KIRC patients.

Previous studies have found that CSF2 facilitates tumor progression in malignancies such as bladder, prostate, and gastric cancers by promoting neovascularization within the tumor microenvironment [45–47]. This pro-angiogenic effect is frequently mediated by inflammatory immune cells [48], particularly neutrophils, which play a pivotal role in shaping the TME of tumors such as gastric cancer [49]. Neutrophils exhibit phenotypic plasticity, polarizing into either N1 (anti-tumorigenic) or N2 (pro-tumorigenic) subtypes. This study hypothesizes that CSF2 facilitates renal cancer progression through the polarization of neutrophils toward the tumor-promoting N2 phenotype.

Neutrophils interact closely with T cells to modulate immune responses *in vivo* [50]. For T cells, PD-1 is commonly involved in the immune function [51]. Upon binding to its ligand PD-L1, PD-1 can inhibit normal T-cell function and promote tumor growth, similar to the role of CSF2 [52]. During inflammatory responses, neutrophil-derived ROS can influence T-cell behavior [53]. ROS can also play an anti-tumor or tumor-promoting role [54]. Previous studies have found that luteolin can increase the content of ROS in glioblastoma, affect the function of endoplasmic reticulum and mitochondria, and then improve the level of tumor apoptosis [55]. Western blot analysis in this study revealed a significant upregulation of PD-L1 protein expression in CSF2-treated neutrophils. Flow cytometry further confirmed increased PD-L1 and decreased ROS levels, implying a tumor-suppressive role for ROS in renal cancer. The reduction in ROS also suggests a phenotypic shift from N1 to N2 neutrophils. Additional flow cytometry detected increased CD11b expression and reduced apoptosis in neutrophils following CSF2 treatment. Transwell assays indicated enhanced neutrophil migration. Immunoblotting for autophagy-related proteins – ATG7, Beclin1, and LC-3 – demonstrated that CSF2 significantly promoted autophagic activity. Notably, PD-L1 knockout reversed this effect, indicating that PD-L1 is a crucial mediator linking CSF2 signaling to autophagy induction. Combining CSF2 with autophagy inhibitors further revealed mechanistic insights: CSF2-enhanced migration was suppressed, and apoptosis was increased upon autophagy inhibition. Interestingly, 3-MA, an early-stage autophagy inhibitor, effectively blocked cell migration, whereas CQ, which targets late-stage autophagy, did not. This suggests that CSF2-driven migration relies on early autophagy events, and interruption at the initiation stage (via PI3K inhibition by 3-MA) more effectively disrupts pro-migratory signaling [29,30].

The recent advancements in AlphaFold, a deep learning-based model for protein structure prediction, have significantly transformed molecular biology and drug discovery. A recent scientometric analysis has quantitatively demonstrated the rapid growth and broad influence of AlphaFold-related research, identifying “structure prediction,” “artificial intelligence,” “drug discovery,” and “molecular dynamics” as core hotspots driving the field forward [56]. In this context, AlphaFold’s predictive capabilities may provide critical structural insights into proteins such as PD-L1, CSF2 receptor (CSF2R), and autophagy regulators (e.g., LC-3, Beclin1). For example, AlphaFold can identify key binding interfaces for drug development, model conformational changes upon ligand binding, and predict phosphorylation sites involved in protein function. Structural modeling of CSF2–CSF2R and PD-L1–autophagy protein interactions could greatly accelerate therapeutic design targeting the CSF2–PD-L1 axis in renal cancer.

However, this study has several important limitations that should be addressed in future research. First, the experiments are primarily based on *in vitro* cell models, lacking validation in animal models or clinical samples, making it difficult to directly translate the conclusions to clinical applications. The reliance on *in vitro* models also limits the ability to capture the complexity of the tumor microenvironment *in vivo*. Future studies should aim to incorporate animal models for *in vivo* validation and clinical samples to enhance the relevance and applicability of the results. Furthermore, the study lacks an in-depth exploration of the molecular mechanisms by which CSF2 modulates the tumor microenvironment, particularly its potential impact on other immune cells. While neutrophils were a focus, other immune components within the tumor microenvironment, such as T cells, macrophages, or dendritic cells, could play critical roles in the immune response [57]. Future research should consider investigating these additional immune cells to fully understand the interplay between CSF2, neutrophils, and other immune cells, and to identify potential therapeutic targets within the tumor microenvironment. Future research should also focus on increasing the sample size, incorporating animal models for *in vivo* validation, and further exploring the multi-cellular interactions and molecular mechanisms by which CSF2 regulates the tumor microenvironment, which may reveal new therapeutic targets.

In summary, this study confirmed that CSF2 induces N2 phenotype neutrophil polarization via the PD-L1 pathway, providing a new target and experimental basis for developing renal cancer therapies, which has significant clinical implications.
